# Examination of Social Participation in Older Adults Undergoing Frailty Health Checkups Using Deep Learning Models

**DOI:** 10.3390/geriatrics10050124

**Published:** 2025-09-12

**Authors:** Yoshiharu Yokokawa, Keisuke Nakamura, Tomohiro Sasaki, Shinobu Yokouchi, Fumikazu Kimura

**Affiliations:** 1 School of Health Sciences, Faculty of Medicine, Shinshu University, Matsumoto 390-8621, Japan; keipons08@shinshu-u.ac.jp (K.N.); kimura_f@shinshu-u.ac.jp (F.K.); 2 Health Promotion Division, Matsumoto City Hall, Matsumoto 390-8620, Japan; tomohiro_sasaki@city.matsumoto.lg.jp (T.S.); shinobu_yokouchi@city.matsumoto.lg.jp (S.Y.)

**Keywords:** older adults, machine learning, deep neural network, social participation, information-collection ability

## Abstract

**Background/Objectives**: Frailty in older adults limits social participation. We aimed to predict social participation in older individuals undergoing frailty health checkups using three machine learning (ML) models and identify key predictive factors through deep neural network (DNN) analysis. **Methods**: Overall, 301 older individuals were enrolled; 295 were included in the final analysis. The survey measured 18 attributes, including demographic, physical, cognitive, and social factors. Logistic regression (LR), nonlinear support vector machine (NLSVM), and DNN were used for prediction, with precision, accuracy, sensitivity, specificity, F1 score, and area under the curve (AUC) calculated as evaluation metrics. **Results**: Among 295 participants, 236 (80%) engaged in social activities, whereas 59 (20%) did not. The three models demonstrated complementary strengths: DNN provided the most balanced performance with superior sensitivity for detecting social participants; NLSVM showed the best overall discriminative ability but with higher false positive rates; and LR achieved the highest precision for correctly identifying participants but missed detecting social participants. AUC values ranged from 0.776 to 0.795 across models, indicating moderate discriminative performance. Contribution analysis revealed information-collection ability as the strongest predictor of social participation, followed by walking speed and number of cohabitants. **Conclusions**: ML models achieved moderate discriminative performance for predicting social participation among frailty-screened older adults. The DNN provided the most balanced performance. Each model exhibited distinct characteristics suitable for different screening purposes, with information-collection ability emerging as a key factor. The findings suggest that models must be carefully selected based on specific community health screening objectives.

## 1. Introduction

Social participation among older adults is linked to health maintenance and promotion, and good health status is confirmed to promote social participation, indicating a bidirectional relationship [1,2]. Factors promoting social participation include employment motivation, sense of purpose, and health status, whereas deterioration of mental health, bereavement, illness, and disability are reported to limit social participation [3,4,5,6,7,8,9,10,11].

Frailty, in particular, is an important factor that hinders the physical and mental functions and activities of older adults, limiting social and daily life activities [12,13]. Multiple epidemiological studies have revealed that the proportion of older adults diagnosed with frailty and prefrailty exceeds approximately 40% [14,15], making this figure an issue that cannot be overlooked in an aging society. Continuing social participation is considered extremely important for maintaining health despite frailty risk [16]. Additionally, early identification of frailty is crucial for timely intervention and optimal management strategies [17].

Previous studies have clarified that various factors are independently associated with social participation in healthy older individuals [18]; however, the interaction among environmental, social, and lifestyle factors for maintaining social participation among older individuals at risk of frailty has not been fully elucidated [19,20]. Traditional statistical approaches present limitations in comprehensively analyzing these complex interrelationships [21].

Machine learning (ML) is a computational technique capable of identifying potential patterns from complex datasets and constructing high-precision predictive models [22,23]. It consists of diverse algorithms that autonomously learn from data and convert it into useful knowledge while minimizing human intervention [24]. Deep learning models, in particular, excel at detecting nonlinear relationships and complex interactions that traditional statistical methods cannot capture [25,26].

However, to date, no studies have comprehensively examined physiological, anthropometric, environmental, social, and lifestyle factors associated with social participation in older adults undergoing frailty health checkups using deep learning approaches. The application of ML and deep learning models may improve prediction accuracy and enhance the reliability of insights by enabling comparisons across models.

Therefore, this study aimed to identify predictive factors for social participation in older adults undergoing frailty health checkups using deep learning models and assess their relative importance. The findings are expected to support the development of effective intervention strategies to promote social participation and optimize frailty prevention and management programs in this population.

## 2. Materials and Methods

### 2.1. Participants

A total of 301 older adult residents aged ≥65 years from Matsumoto City, Nagano Prefecture, who participated in local exercise classes, responded to the 2022 frailty health check project recruitment. This city-led initiative conducts frailty assessments using questionnaires and evaluates physical functions, such as walking speed and grip strength, to identify frail individuals, recommend treatment, and prevent prefrailty.

### 2.2. Measurement Items

Demographic items (sex, age, subjective economic status, years of education, number of cohabitants), number of diseases, walking speed, frailty category classification, grip strength, history of falls, presence of health checkups, sleep duration, employment status, cognitive function, health literacy, instrumental activities of daily living (IADL), total medical expenses in 2022, social participation ability, and information-collection ability were measured as reported in previous studies [27] (Table 1).

Walking speed was assessed by measuring the time required to walk a 5 m straight path at a comfortable pace.

Frailty classification followed category 3 of the revised Japanese version of the Cardiovascular Health Study criteria, whereby individuals meeting three or more of the five criteria were classified as frail, those meeting one or two as prefrail, and those meeting none as robust [11].

Cognitive function was evaluated using 3 items from the 25-item basic checklist, a screening tool designed to assess daily living functions [28]. The questions were as follows: (1) Do people around you say you often forget things, such as asking the same question repeatedly? (2) Do you look up phone numbers and make calls by yourself? and (3) Do you sometimes not know what month or day it is? Cognitive decline was defined as one or more of these items being applicable.

Health literacy was assessed using the communicative and critical health literacy scale, which consists of five items [29]. This scale evaluates the individual’s ability to independently seek out and apply information related to illness and health. Participants responded on a 5-point scale ranging from “strongly disagree” to “strongly agree,” with total scores ranging from 5 to 25 points.

IADL was assessed using five subscale items drawn from the Tokyo Metropolitan Institute of Gerontology Index of Competence. Each item was assigned 1 point for a “yes” response and 0 points for a “no” response, resulting in a total score ranging from 0 to 5 points [30].

The ability to engage in social participation was assessed using a subscale comprising 4 items from the Japan Science and Technology Agency Index of Competence (JST-IC) version of the 16-item Activity Ability Index, which measures the capacity to participate in and fulfill roles within community activities [31]. Each item was assigned 1 point, allowing for a total score ranging from 0 to 4 points. Social participation was considered to be absent when the score was 0 and present when it was ≥1 point. The questions were as follows: (1) Do you participate in local festivals or events? (2) Are you active in neighborhood or community associations? (3) Can you take on roles or positions in community or group activities? and (4) Do you engage in service or volunteer activities? Social participation was prespecified as binary (0 for non-participants; ≥1 for participants) to reflect the program’s screening objective of reliably flagging non-participants for outreach. Further, with the use of our small and imbalanced sample, collapsing data to 0 vs. ≥1 reduces model complexity and variance, thereby improving statistical stability and reproducibility. This decision was made a priori to match operational workflows in community services. From a measurement standpoint, the 4-item scale makes within-participation gradations (1–4) susceptible to item-level error, whereas 0 denotes a qualitatively distinct absence of participation. When using small, imbalanced samples, collapsing data to 0 vs. ≥1 reduces the number of parameters and helps prevent overfitting, improving robustness and reproducibility. Operationally, frontline services require binary triage under asymmetric error costs, and the binary endpoint aligns predictions with actionable decision-making.

The ability to collect information was similarly evaluated using a 4-item subscale from the JST-IC version of the 16-item Activity Ability Index, focusing on the capacity to independently gather and apply information to daily life [31]. The four questions were assigned 1 point for “yes” responses and 0 for “no” responses, with possible total scores ranging from 0 to 4 points. Participants were asked the following questions: (1) Are you interested in foreign news and events? (2) Can you judge the credibility of health-related information? (3) Do you appreciate art, movies, or music? and (4) Do you watch educational or cultural programs?

### 2.3. Analysis Method

**Figure 1:**
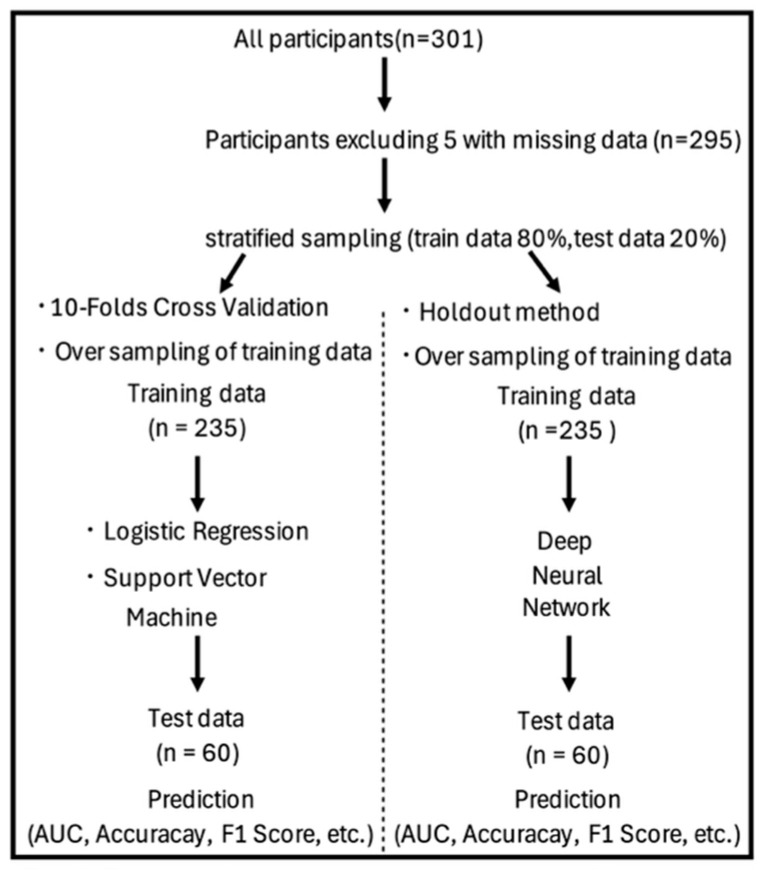
Flowchart describing the overview of the support vector machine and deep neural network approach.

The dataset showed class imbalance, with 236 individuals who participated in social activities (80%) and 59 who were non-participants (20%) (Table 1). Predictive analyses were performed using ML techniques, with all 18 measured items as explanatory variables and the presence or absence of social participation as the outcome variable. The holdout validation method was used in the DNN, and the data was randomly split into a training set (80%, 235 individuals) and a test set (20%, 60 individuals) (Figure 1).

#### 2.3.1. Data Splitting and Handling of Class Imbalance

We partitioned the dataset into training (80%; n = 235) and test (20%; n = 60) sets using a stratified split based on the outcome (social participation: participants vs. non-participants), such that each subset preserved the original prevalence (approximately 80% participants and approximately 20% non-participants) and ensured representation of the minority class. A fixed random seed was used for all random operations to ensure reproducibility. To mitigate class imbalance during model training, we duplicated each minority-class instance once within the training set, thereby doubling the number of non-participants. No resampling was applied to the test set, which retained the original prevalence to provide unbiased performance estimates.

#### 2.3.2. Logistic Regression (LR) and Nonlinear Support Vector Machine (NLSVM)

LR uses a simple classification approach that uses a linear equation with one or more independent variables to predict a binary dependent variable. The predicted values are mapped to probabilities using the sigmoid function.

The support vector machine (SVM), developed by Cortes and Vapnik [32], is a supervised ML algorithm that identifies a boundary, known as a hyperplane, which maximally separates groups. In this study, we employed an NLSVM, an extension of the basic SVM that is capable of addressing nonlinear boundaries through the use of kernel functions. NLSVM is particularly suited for analyses involving small datasets and performs effectively in high-dimensional feature spaces. To evaluate the model’s generalization performance, 10-fold cross-validation (10-FCV) was conducted. In 10-FCV, the dataset was randomly divided into 10 subsets using stratified sampling, ensuring that each fold maintained the same participation: non-participation ratio as the original dataset, with nine subsets used for training and the remaining 1 subset used for validation in each iteration. Classification accuracy was computed as the median across the 10 folds. Model performance was further assessed by calculating the area under the receiver operating characteristic (ROC) curve (AUC) using the predicted probabilities from the NLSVM. All analyses were performed using the R4.5.0 software.

#### 2.3.3. Deep Neural Network (DNN)

For comparisons with the previously described NLSVM, we conducted an additional analysis using a different classification algorithm, a DNN [24]. In this analysis, missing data were excluded, yielding a final dataset of 295 samples. The dataset was divided into a training set (235 samples, used for model development and validation) and a test set (60 samples, used for evaluation) using stratified sampling to preserve the original participation: non-participation ratio in both subsets. During the implementation of the holdout validation method, oversampling was performed to address class imbalance in the DNN training data. To mitigate model bias arising from differences in the numerical scales of the variables, min-max scaling was applied, whereby each variable was linearly transformed so that its minimum and maximum values were set to 0 and 1, respectively, with intermediate values proportionally adjusted within this range. Ten percent of the training data was reserved as a validation subset for tuning the architecture (i.e., hyperparameters) of a fully connected DNN. The primary hyperparameters—including the number of hidden layers, number of neurons per layer, activation functions, number of training epochs, and batch size—were optimized through iterative training. Model performance was assessed by calculating the root mean square error (RMSE) between the predicted and actual values on a validation set, separated from the training set. Among the 10 trained models, the 3 models with the lowest RMSE on the validation set were selected. The DNN architecture and learning details for the three optimal models, respectively, were as follows: the number of hidden layers were 10, 8, and 4; the number of neurons in hidden layers were 84, 96, and 29; the dropout rate for the input layer and for layers other than the input layer were 0; the activation function for the output layer was sigmoid; the activation function for layers other than the output layer was rectified linear unit; the loss function was mean squared error; the optimization method was Adam; and the number of epochs until learning stopped were 7887, 3016, and 7888. Their prediction outputs were then combined by simple averaging to construct an ensemble model, which was subsequently used to perform ROC analysis to evaluate predictive accuracy. To assess the contribution of each explanatory variable to the prediction, a contribution analysis was carried out on the ensemble model that showed the lowest RMSE and highest coefficient of determination (R^2^). This analysis applied the partial derivative method proposed by Gevrey et al. to quantify the positive and negative influences of each explanatory variable on the target variable [33]. Consequently, the most influential explanatory variables were identified.

All analytical procedures were performed using the cloud-based analysis platform Multi-Sigma^®^ (AIZOTH Inc., Tsukuba, Japan) [34].

To compare the predictive performance, precision, accuracy, sensitivity, specificity, F1 score, and AUC were calculated for the LR, NLSVM, and DNN models. The F1 score balances recall and precision, whereas AUC measures the ability of the model to distinguish between classes. In principle, all three models are capable of modeling nonlinear relationships and thus can capture complex phenomena. Therefore, the relative predictive performance of these models must be evaluated based on these metrics using actual data.

### 2.4. Ethical Considerations

This study was approved by the Matsumoto City Hospital Ethics Committee (Research Number: 03–5). As this was a preventive health check program for frailty risk conducted as part of Matsumoto City’s health policy, informed consent was obtained through an opt-out protocol. Information regarding the opportunity to opt out was provided in advance through guidance documents distributed to potential participants and was also included in the explanatory documents provided during the frailty health examination. Participants were also informed that they could withdraw from the study at any time.

## 3. Results

### 3.1. Basic Information of Participants

A total of 301 individuals participated in the 2022 frailty health check. Of these, 295 participants (98%) were included in the analysis after excluding those with missing data. Among them, 236 (80%) and 59 (20%) participated and did not participate in social activities, respectively.

### 3.2. Characteristics of Social Participation and Analysis of ML Models

In the group with social participation, walking speed, IADL score, health literacy, and information-collection ability were higher than those in the group without social participation (Table 1). Table 2 presents the comparison of discriminative performance of the three ML models. The DNN demonstrated the highest sensitivity (0.833) and F1 score (0.625), with balanced performance across metrics. The NLSVM achieved the highest AUC (0.795) but showed the lowest precision (0.438), indicating a tendency to generate false positives. The LR model exhibited the highest precision (0.894) and specificity (0.833) but demonstrated the poorest overall performance with the lowest F1 score (0.519) due to limited sensitivity (0.583). All models showed moderate discriminative ability with AUC values ranging from 0.776 to 0.795. In the contribution analysis of social participation based on the DNN, no variables showed a positive contribution rate exceeding twice the average contribution rate of 6.6% (Table 3). The highest positive contribution was 12.28% for information-collection ability, followed by 4.54% for walking speed, and 3.98% for sleep duration.

## 4. Discussion

This study explored the application of ML approaches for identifying factors associated with social participation among community-dwelling older adults. The analysis revealed a substantial disparity in social engagement patterns within the study population, with the majority actively participating while a minority remained disengaged, creating a class imbalance that fundamentally influenced algorithmic behavior and model performance characteristics. Among the three ML models evaluated, each exhibited distinct performance profiles that reflected inherent algorithmic priorities and varying approaches to the sensitivity-specificity trade-off dilemma. The DNN emerged as the most balanced classifier, demonstrating superior ability to correctly identify both participants and non-participants with consistent discriminative performance across evaluation metrics. In contrast, the NLSVM exhibited the strongest overall discriminative capacity but at the cost of generating excessive false positive classifications, suggesting heightened sensitivity to minority class detection. The LR model demonstrated exceptional precision in identifying true participants but suffered from limited sensitivity, frequently failing to detect individuals who actually engage in social activities. The contribution analysis revealed information-collection ability as the variable with the highest contribution value, though no variables demonstrated statistically significant predictive strength, with walking speed and sleep duration showing comparatively modest contributions among the evaluated factors. The moderate discriminative performance across all models underscores both the inherent complexity of predicting social behavior and the methodological challenges associated with imbalanced classification problems in behavioral health research.

Previous studies have employed multiple ML analyses to assess frailty and social participation in older adults [26,35,36,37,38], reporting AUC values of 0.7–0.9, with some longitudinal studies achieving AUCs >0.9 [39]. Compared with these studies, in our study, the models demonstrated moderate performance metrics, though the class imbalance issue requires careful consideration. In comparison, the present findings indicate a model with adequate indicator values for identifying social participation.

Information-collection ability showed the highest contribution to social participation prediction. Previous studies reported that information-collection ability, together with social participation ability, forms part of the JST Index of Competence, and a correlation was observed [31]. A strategy to sustain and promote social participation among older adults involves the creation of routine tasks, one of which is an introspective, inward-focused approach. Examples include maintaining a positive mindset, cultivating ongoing relationships linked to identity, and engaging in activities that preserve one’s social role [40]. Information-gathering activities are thought to support such strategies. The definition of social participation as the outcome variable refers to engagement in semi-public community events, such as festivals or neighborhood association activities, and it can be understood as participation in socially contributive roles accompanied by a certain level of responsibility and obligation. If “no” responses are given for all four question items, the level of involvement in such activities is considered very low.

The walking speed of older adults is associated with life prognosis and serves as an important indicator of health status [41]. Good health status is considered to facilitate social activities, including productivity [42]. However, as these are not proven causal relationships, caution is warranted in their interpretation.

Conversely, for health literacy, the negative contribution was greater than the positive contribution. Older adult health literacy is considered to be associated with health indicators, such as engagement in health behaviors, and is linked to increased motivation for social participation [43]. Generally, high health literacy promotes the adoption of preventive health behaviors. However, the reason for older individuals with higher health literacy in this study being less likely to participate in social activities remains unclear. The unexpected negative contribution of health literacy requires careful interpretation and warrants further investigation through subgroup analyses and longitudinal studies to understand potential confounding factors and causal relationships. The type of social participation examined here involved roles required or expected by the community, such as serving as an officer in a neighborhood association. This form of participation may easily elicit a sense of obligation and stress, potentially giving rise to negative emotions. The limited impact of frailty suggests that functional ability, more than physical function, may influence social participation. Frail older individuals who participated in hobby or sports clubs had a 26–28% lower risk of needing care certification and a 25–32% lower risk of death, compared with those not involved in social activities [44]. Longitudinal studies have confirmed that social participation desired by the individual is effective in preventing frailty [45]. However, it is possible that older adults aware of their frailty or prefrailty may withdraw from participation in high social contribution activities, such as volunteering or positions with responsibility. Future studies should investigate the specific circumstances under which health literacy exerts positive versus negative influences on screening participation.

The limitations of this study are as follows. First, it employed a cross-sectional survey design, which does not allow for causal inferences. Future research using longitudinal data will be necessary. Second, there is uncertainty regarding the adequacy of the independent variables selected to predict social participation, and it remains possible that relevant variables were overlooked. The inclusion of unverified variables may offer new perspectives. Third, our sample consisted older residents in the area who regularly participated in exercise classes, representing a group of health-conscious and physically active seniors. This sampling may limit the generalizability of the findings to older adults who are less physically active or socially engaged. Fourth, the dataset could not eliminate class imbalance, which might have affected the model’s performance. Future research should address class imbalance through appropriate methods (e.g., synthetic minority oversampling technique, weighted loss functions) and better contextualize the usefulness of complex ML approaches, including external validation. Finaly, we acknowledge that a binary classification approach may overly simplify the ordinal nature of social participation, and ordinal regression approaches might be beneficial in future studies.

Several important challenges must be resolved to utilize the ML models from this study in actual support services for older adults. First, appropriate threshold setting is necessary to properly adjust the trade-off between sensitivity and specificity, considering the urgency of intervention and resource constraints. Second, to improve prediction accuracy, incorporation of additional predictive factors (social networks, geographical factors, past participation history, etc.) should be considered. Third, implementation of multicenter collaborative research is essential to verify generalization performance in older populations with different regional and cultural backgrounds. Finally, development of decision support systems for medical and health professionals is required to utilize prediction results in actual intervention planning.

## 5. Conclusions

ML models demonstrated moderate discriminative performance for predicting social participation among frailty-screened older adults, with the NLSVM achieving the highest AUC and the DNN demonstrating the most balanced performance with the highest F1 score and sensitivity. Information-collection ability emerged as the strongest predictor across models. The variable performance characteristics of the models suggest the need for careful model selection based on specific screening objectives, with the DNN offering balanced sensitivity and specificity for comprehensive assessment, the NLSVM providing superior overall discriminative ability despite lower precision, and the LR model delivering high precision for targeted identification of social participants.

## Figures and Tables

**Table 1:** Descriptive data of subjects.

	Social Participation		
Characteristic	No (N = 59)	Yes (N = 236)	*p*-Value
Sex (female)	50 (85%)	191 (81%)	0.5
Age	81 (78, 85)	79 (73, 83)	0.042
Walk speed (m/s)	1.09 (0.96, 1.31)	1.22 (1.04, 1.38)	0.012
Grip strength (kg)	23 (18, 26)	23 (20, 26)	0.083
Frailty category classification			0.051
Robust	34 (58%)	149 (63%)	
Prefrail	18 (31%)	55 (23%)	
Frail	7 (12%)	32 (14%)	
Number of diseases (n = 0)	45 (76%)	186 (79%)	0.6
Year of education			0.6
9 year	11 (19%)	37 (16%)	
12 year	37 (63%)	135 (57%)	
15 year	9 (15%)	53 (22%)	
others	2 (3.4%)	11 (4.7%)	
Number of cohabitants			0.7
Couple	14 (24%)	74 (31%)	
Alone	16 (27%)	65 (28%)	
Couple and child	8 (14%)	28 (12%)	
Three generation household	6 (10%)	18 (7.6%)	
Child	12 (20%)	33 (14%)	
Others	3 (5.1%)	18 (7.6%)	
Subjective economic status			0.8
Can afford to live	4 (6.8%)	14 (5.9%)	
Can afford to live a little	10 (17%)	35 (15%)	
Neither	37 (63%)	163 (69%)	
Can not afford to live a little	6 (10%)	18 (7.6%)	
Can not afford to live	2 (3.4%)	6 (2.5%)	
Employment status (yes)	6 (10%)	32 (14%)	0.5
Health checkups (yes)	48 (81%)	195 (83%)	0.8
Sleeping duration(h)	7.00 (5.25, 8.00)	7.00 (6.00, 8.00)	0.6
History of falls (yes)	10 (17%)	49 (21%)	0.5
Cognitive decline (yes)	55(93.2%)	223(94.5%)	0.3
IADL	5.00 [4.00, 5.00]	5.00 [5.00, 5.00]	<0.001
Health Literacy	18 (16, 20)	20 (18, 21)	0.002
Information collection ability	2.00 [0.00, 3.00]	4.00 [3.00, 4.00]	<0.001
Medical cost in 2022	267,540 (137,235, 433,490)	236,790 (96,868, 423,120)	0.3
Median (IQR); n (%)			
Wilcoxon rank sum test; Pearson’s Chi-squared test; Fisher’s exact test			

**Table 2:** Comparison of discrimination for social participation.

	Precision	Accuracy	Sensitivity	Specificity	F1 Score	AUC
LR	0.894	0.783	0.583	0.833	0.519	0.776
NLSVM	0.438	0.767	0.583	0.812	0.5	0.795
DNN	0.5	0.8	0.833	0.792	0.625	0.788

LR: Logistic regression; NLSVM: Nonlinear support vector machine; DNN: Deep neural network.

**Table 3:** Factor analysis by Deep Neural Network.

	Contribution [%]	Contribution_Negative [%]	Contribution_Positive [%]
Information-collection ability	12.45	0.17	12.28
Walk speed	5.78	1.24	4.54
Sleeping duration	5.98	1.99	3.98
Number of cohabitants	5.16	1.31	3.85
IADL	5.95	3.14	2.81
Cognitive decline	5.65	2.95	2.7
Health Literacy	8.04	5.44	2.61
Grip strength	6.31	3.74	2.56
History of falls	3.85	1.46	2.39
Number of diseases	4.3	2.19	2.11
Health checkups	3.21	1.2	2.02
Year of education	4.82	2.92	1.9
Employment status	3.75	2.02	1.73
Age	5.19	3.51	1.68
Medical cost in 2022	5.33	3.97	1.35
Sex	4.28	2.95	1.33
Subjective economic status	5.46	4.27	1.19
Frailty category classification	4.5	3.58	0.93

## Data Availability

The data that support the findings of this study are available on request from the corresponding author. The data are not publicly available due to privacy or ethical restrictions.

## Abbreviations

The following abbreviations are used in this manuscript:

NLSVM	Nonlinear support vector machine
DNN	Deep neural network
ML	Machine learning
LR	Logistic regression
AUC	Area under the curve
IADL	Instrumental activities of daily living
10-FCV	10-fold cross-validation
SVM	Support vector machine
RMSE	Root mean square error
